# Paraganglioma of the tongue with SDHB gene mutation in a patient with Graves’ disease

**DOI:** 10.1002/ccr3.2065

**Published:** 2019-03-02

**Authors:** Marcela Adriana Duran Alvarez, Juan Jose Tavarez Rodriguez, Mercedes Robledo

**Affiliations:** ^1^ Department of Pathology Hospital de Medina del Campo Valladolid Spain; ^2^ Department of Otorhinolaringology Hospital de Medina del Campo Valladolid Spain; ^3^ Hereditary Endocrine Cancer, Human Cancer Genetics Programme Spanish National Cancer Centre (CNIO) Madrid Spain

**Keywords:** Graves’ disease, mutation, paraganglioma, SDHB, thyroid, tongue

## Abstract

We report a case of an apparently sporadic paraganglioma of the tongue with a germ‐line mutation in a female patient with asymptomatic Graves’ disease. The tongue is an unusual primary location. Genetic testing is mandatory in all cases. Thyroid gland dysfunction and autoimmune phenomena could be associated with some paragangliomas.

## 1 INTRODUCTION

Paragangliomas (PGLs) are uncommon neuroendocrine tumors arising from the neural crest‐derived paraganglia of the autonomic nervous system, including the adrenal medulla, represented by pheochromocytoma (PCC). Most of the sympathetic‐related PGLs are chromaffin and functioning tumors associated with epinephrine and/or norepinephrine release. Conversely, most of the parasympathetic PGLs are nonchromaffin and nonfunctioning tumors although some of them can produce dopamine.^1^, ^2^ Outside the adrenal gland, the most frequent location is the head and neck area with a predominance in female patients. These PGLs are usually parasympathetic tumors arising from the carotid body, jugulotympanic paraganglia, and the ganglion nodosum of the vagus nerve**.** 1, 3, 4 Other rarer sites of involvement are the thyroid gland, larynx, nose, orbit and along the hypoglossal nerve, but occurrence in oral structures is extremely uncommon.^3^, ^4^ To the best of our knowledge, only four PGLs of the tongue were reported previously.^2^, ^4^, ^5^

Most PGLs follow a benign clinical course. Estimated rates of malignancy were about 10%; however, long‐term follow‐up and advances in genetics confirmed that the real incidence of malignancy is higher.^6^ Currently, there are no reliable histological criteria to predict the biological behavior; hence, malignancy is defined by the presence of metastases. Bone, lymph nodes, lung, and liver are the most frequent metastatic locations.^6^

These tumors carry the highest degree of heritability among human neoplasms. 40% of PGLs harbor a germline mutation and an additional 20%‐30% a somatic mutation.^7^, ^8^ Up to 14% of apparently sporadic tumors carry occult germline mutations.^7^, ^8^ Hereditary syndromes, such as multiple endocrine neoplasia (MEN) type 2A and 2B, von Hippel‐Lindau (vHL), and neurofibromatosis type 1 (NF‐1) are associated with the development of PGLs. In addition, hereditary susceptibility also includes five familial PGLs syndromes caused by mutation in the succinate dehydrogenase genes (collectively SDHx) that can be screened by immunohistochemistry and identified by sequencing analysis.^9^, ^10^ SDHx mutations represent almost 30% of mutated PGLs. Other genes associated with individual hereditary PCC/PGL syndromes are MAX, TMEM127, KIF1B, EGLN1, and FH, with a prevalence of less than 2% for each gene.^1^

Multinodular goiter, Graves’ disease, and other autoimmune disorders have been described in patients with functioning PCC^11^, ^12^, ^13^, ^14^, ^15^, ^16^, ^17^ and nonfunctioning PGLs of the head and neck including one case located in the tongue.^4^

## 2 CASE REPORT

### 2.1 Clinical history

**Figure 1:**
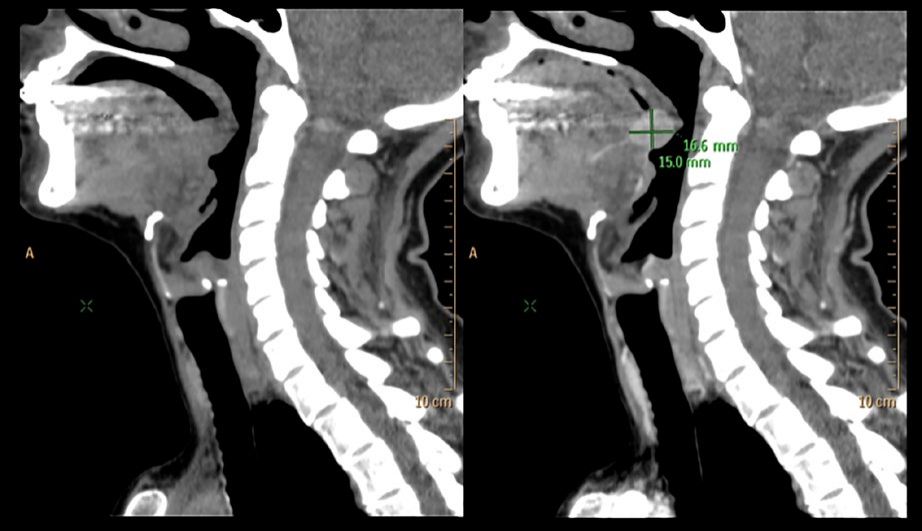
Head and Neck CT scan showing a nodule in the posterior aspect of the tongue (left) with contrast enhancement (right)

**Figure 2:**
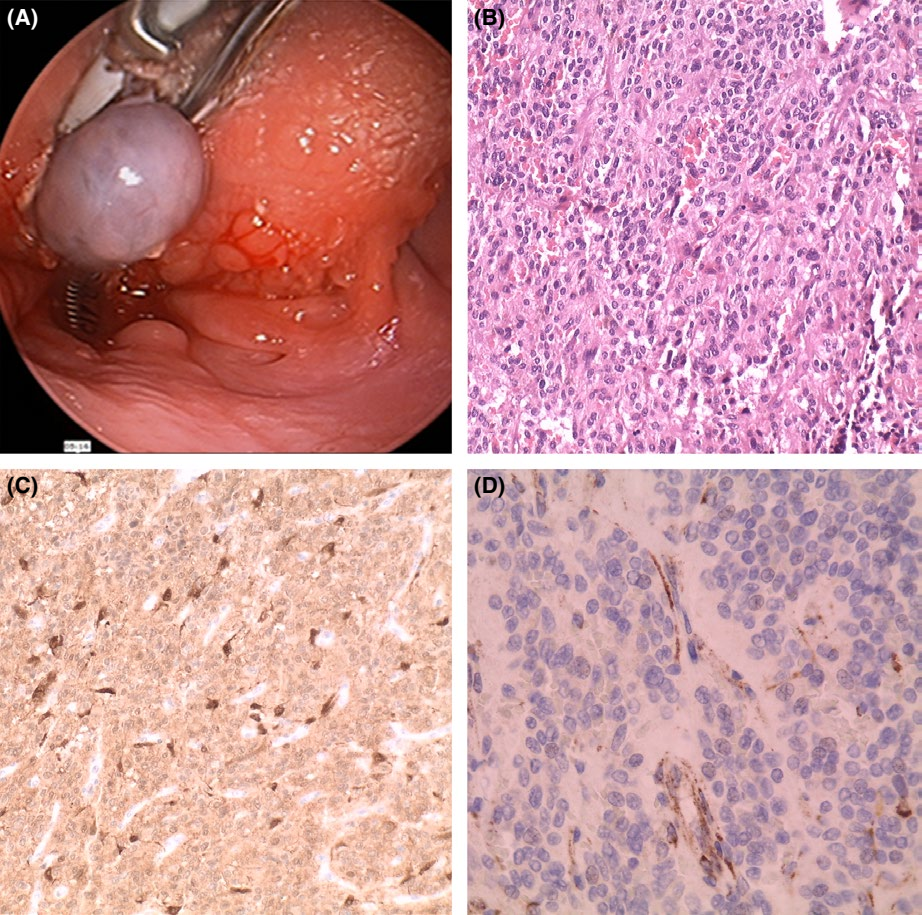
A, Transoral resection of the nodule. B, Panoramic of the tumor (H&E × 200). C, Zellballen appearance with S‐100 positive sustentacular cells (×200). D, Immunohistochemistry for SDHB is negative in tumor cells and positive in endothelium as an internal control (×400)

A 53‐year‐old female was referred by her physician to the Department of Otorhinolaryngology with a swelling in her tongue that she noted after she choked on a piece of food. Biochemical signs of hyperthyroidism (TSH: 0.005 mU/mL, normal ranges: 0.27‐4.20; plasma‐free T4: 2.75 ng/mL, normal ranges: 0.93‐1.71; plasma‐free T3: 5.36 pg/mL, normal ranges: 2.04‐4.40) were detected in a routine analysis three months before she choked. Positive antinuclear antibodies (ANA: positive in 1/40), antithyroid peroxidase (anti‐TPO: 127, 8 U/ml, normal ranges: 0.00‐34.00), anti‐TSH receptor (2.22 U/L, normal ranges: 0.00‐1.75), and antithyroglobulin (anti‐TGB: 11.98 U/ml, normal ranges: 0.00‐115.00) were also detected. Serum calcium was normal (9.95 mg/dl, normal ranges: 8.6‐10.4).These features are consistent with Graves’ disease, although the patient had none of the classical symptoms of this disease. Her familial history was unremarkable, without antecedents of endocrinal or genetic diseases. On physical examination, a nodule bulging under the posterior aspect of the tongue was observed. Neither dysgeusia nor dysarthria were detected. Head and neck computed tomography (CT) scan evidenced a nodule at the base of the tongue with intense enhancement after contrast administration, suspicious for a hemangioma (Figure 1). No other masses were detected on the head and neck area. Thyroid gland ultrasound identified two nonspecific millimetric nodules in a normal sized gland. Radioiodine scan showed an orthotopic thyroid gland with a diffusely increased uptake. After the diagnosis, a thoracoabdominal CT scan ruled out adrenal or extra‐adrenal masses. The patient underwent an endoscopic transoral resection of the lingual nodule (Figure 2A). Healing was uneventful.

### 2.2 Materials and methods

The surgical specimen was fixed in 10% buffered formalin for routine histology and immunohistochemistry. The latter was performed using the avidin‐biotin technique. Tested antibodies (all from Dako, Glostrup, DK‐Denmark) included chromogranin A, neuron‐specific enolase (NSE), S‐100 protein, CK AE1/AE3, smooth muscle antigen (SMA), CD‐31, carcinoembryonic antigen (CEA), thyroglobulin, calcitonin, TTF‐1, HMB‐45, and Ki67. Immunohistochemistry and sequencing analysis for SDHx mutations were performed in another center using anti SDH B rabbit polyclonal antibody (Sigma‐Aldrich Corp). SDHA was also tested on tumor sample as previously described.^9^ For molecular analysis, Sanger and next‐generation sequencing (NGS) were applied. It was used a TruSeq Custom Amplicon 1.5 Kit (Illumina, San Diego, CA) to study DNA from tumor and blood samples as previously described.^10^

### 2.3 Pathologic findings

The surgical specimen consisted of a rubbery brown nodule, measuring 17 × 15 × 15 mm, with well circumscribed borders, partially covered by an intact mucosa. Microscopic examination revealed a highly vascularized tumor composed of nests of uniform cells with finely granular cytoplasm and round nuclei without atypia, mitosis, or necrosis. These cells expressed chromogranin A and NSE. A second population of S‐100 positive sustentacular cells were detected at the periphery of the nests with the classical “zellballen” appearance described in paraganglioma (Figure 2B,C). The tumor was focally transected by surgical margins. Other markers tested for differential diagnosis are summarized in Table 1. Ki 67 showed a very low proliferation rate (<1%). Other potentially associated tumor components derived from the neural crest, such as ganglioneuroma and melanocytes, were not identified. Immunohistochemistry for SDHB was negative **(**Figure 2D) while SDHA was positive. This result suggested the presence of a mutation in any of the SDHx genes but SDHA. Mutational analysis confirmed a c.689G>A/p.Arg230His mutation in SDHB gene.

**Table 1:** Immunohistochemistry in differential diagnosis

Marker	Result	Interpretation
Chromogranin A	Positive	Favor paraganglioma
NSE	Positive	Favor paraganglioma
TTF‐1	Negative	Exclude thyroid origin, other neuroendocrine tumors
Thyroglobulin	Negative	Exclude thyroid origin
Calcitonin	Negative	Exclude medullary carcinoma
CEA	Negative	Exclude medullary carcinoma
CD31	Negative	Exclude hemangioma
SMA	Negative	Exclude glomus tumor
Cytokeratins	Negative	Exclude carcinoma, other neuroendocrine tumors, and thyroid adenoma
S‐100	Positive sustentacular cells	Classical paraganglioma

## 3 DISCUSSION

### 3.1 Primary vs metastatic PGL

The tongue is richly endowed with nerve fibers, and some of them are autonomic with minor paraganglia.^18^ The tongue is an unusual primary site for PGLs**. **Despite the statement that metastasis of paraganglioma should be diagnosed only if the affected organs lack paraganglia, we consider that any organ can be involved by metastasis; therefore, we ruled out a PGL in other primary locations.

### 3.2 Catecholamines in diagnosis and follow‐up

At least some PGLs of the head and neck produce dopamine.^1^ Biochemical diagnosis for this type of PGLs is best achieved by measurement of plasma‐free methoxytyramine.^1^ Some adrenal PCC and extra‐adrenal PGLs are nonfunctioning tumors, either primarily or after a metabolic change along the pathway of dedifferentiation.^1^ This fact reveals that normal levels of catecholamines or metanephrines should be taken with caution. In our case, as the diagnosis of paraganglioma was unexpected, catecholamines were investigated after surgery and they were in normal **l**evels**.** Methoxytyramine test was unavailable in our center.

### 3.3 Genetics provides relevant biological information that histopathology cannot provide

Features such as young age at onset, bilateral and multiple tumors suggest an inherited background. Our case is an example of an apparently sporadic PGL located in an unusual site, and this fact prompted the search for mutations. It was identified a germline mutation in SDHB gene that has been described in Mediterranean population carrying a 45% of malignancy risk.^19^ Germline mutations in SDHx are inherited in an autosomal dominant manner with an age‐related penetrance.^1^, ^7^, ^8^ Genetic testing should be performed in all PGLs.

### 3.4 Metabolomics and future directions

More than half of the PGLs susceptibility genes encode enzymes involved in the Krebs cycle. SDH is also known as the complex II of the oxidative chain. Five subunits (SDHA, SDHB, SDHC, SDHD, and SDHAF2) are the components of this complex located in the inner mitochondrial membrane. SDH catalyzes the oxidation of succinate into fumarate within the Krebs cycle. The loss of SDH activity leads to a massive accumulation of succinate and high levels of this metabolite cause it to act as an oncometabolite, activating the hypoxia‐induced signaling pathway related to angiogenesis, cell metabolism, and other tumor growth factors.^20^, ^21^, ^22^ In addition, succinate can also promote a particular CpG island methylation phenotype (CIMP) by impairment of histone demethylation.^21^ Metabolomics allowed to group PGLs into two clusters depending on pathogenesis and catecholamine phenotype (Table 2). Cluster 1 PGLs have an “immature” biochemical phenotype while cluster 2 PGLs have a “differentiated” phenotype.^1^, ^7^, ^20^, ^21^ This clustering provided new strategies for treatment of malignant and metastatic PGLs.^22^ The multikinase inhibitor sunitinib has been tested in several patients obtaining partial responses in some cases.^7^, ^8^ At the time of this report, the first clinical trial on sunitinib was ongoing (FIRSTMAPPP).

**Table 2:** Clustering of paragangliomas^1^

Characteristics	Cluster 1 tumors	Cluster 2 tumors
Germline or somatic mutation	vHL – SDHx – HIF 2a – MDH2.FH – PHD2	RET – NF1 – TMEM127 ‐ MAX
Mechanism of pathogenesis	Activation of Hypoxia pathway. Methylation	RAS/MAP KINASE signaling pathway
Biochemical phenotype	Dopaminergic (H&N PGLs) Noradrenergic	Adrenergic Mixed (adrenergic‐noradrenergic)

H&N PGLs, head and neck paragangliomas.

### 3.5 Thyroid dysfunction in PGLs

It should be noted that physiologically catecholamines stimulate thyroxin secretion via beta‐adrenergic receptors while dopamine inhibits thyroxin release. Symptoms of thyroid hyperfunction are quite similar to those of functioning PGLs and PCC. Nevertheless, thyroid dysfunction has also been reported in some nonfunctioning PGLs.^4^The common metabolite in the biosynthesis of catecholamines and thyroid hormones is the aminoacid tyrosine. A potential link between mutated PGL (especially those of the head and neck) and thyroid gland dysfunction requires further investigation. As many other tumors, PCC/PGL could acquire antigenic properties capable to elicit an immune response with potential cross‐reactivity against normal tissues generating autoimmune phenomena, although large series addressing this issue are lacking.

### 3.6 Patient outcome

Our patient refused a specific follow‐up including genetic consultation. After treatment with an antithyroid drug and iodine radiation, the patient became hypothyroid and serum autoantibodies decreased significantly. The latest thoracoabdominal CT scan ruled out the presence of masses or other signs of tumor progression. After 18 months from surgery, the patient is doing well.

## 4 CONCLUSION

Our case illustrates, to the best of our knowledge, the first reported primary PGL of the tongue with a documented mutation in SDHB gene, in a patient with asymptomatic Graves’ disease. The tongue is a primary location for PGLs. The absence of a familial history or other features suggestive of an inherited syndrome should not preclude genetic testing. Thyroid gland dysfunction and autoimmunity could be associated with some nonfunctioning paragangliomas although further investigation is needed.

## CONFLICT OF INTEREST

The authors declare that they have no conflict of interest.

## AUTHOR CONTRIBUTION

MADA: conceived and designed the work, researched, analyzed and interpreted the data and images, drafted, and reviewed the manuscript. JJTR: conceived the work, acquired, analyzed and interpreted the data and images, and reviewed the manuscript. MR: researched, analyzed and interpreted the data, and critically reviewed the manuscript. All authors gave final approval for publication.
